# Hysteresis loops of individual Co nanostripes measured by magnetic force microscopy

**DOI:** 10.1186/1556-276X-6-407

**Published:** 2011-06-02

**Authors:** Miriam Jaafar, Luis Serrano-Ramón, Oscar Iglesias-Freire, Amalio Fernández-Pacheco, Manuel Ricardo Ibarra, Jose Maria De Teresa, Agustina Asenjo

**Affiliations:** 1 Instituto de Ciencia de Materiales de Madrid, CSIC, Madrid, 28049, Spain; 2 Instituto de Ciencia de Materiales de Aragón, Universidad de Zaragoza-CSIC, Zaragoza, 50009, Spain; 3 Departamento de Física de la Materia Condensada, Universidad de Zaragoza-CSIC, Zaragoza, 50009, Spain; 4 Laboratorio de Microscopías Avanzadas (LMA), Instituto de Nanociencia de Aragón (INA), Universidad de Zaragoza, Zaragoza, 50009, Spain

## Abstract

High-resolution magnetic imaging is of utmost importance to understand magnetism at the nanoscale. In the present work, we use a magnetic force microscope (MFM) operating under in-plane magnetic field in order to observe with high accuracy the domain configuration changes in Co nanowires as a function of the externally applied magnetic field. The main result is the quantitative evaluation of the coercive field of the individual nanostructures. Such characterization is performed by using an MFM-based technique in which a map of the magnetic signal is obtained as a function of both the lateral displacement and the magnetic field.

## Background

Magnetization reversal in magnetic nanostructures has been theoretically studied for many years [1] but the possibility of empirical studies of the magnetization reversal in nanosized magnets has renewed the interest of the scientific community [2]. The magnetic nanostructures presented here are valuable candidates for the development of different applications such as high-density and high-speed magnetic information storage, high-speed magnetic random access memories, magnetic sensors and logic devices [3-5]. Such nanostructures present different magnetic behavior as a function of their shape, size, aspect ratio, or distance between adjacent elements [6]. Thus, both the study of individual nanoelements and the understanding of the interaction between adjacent magnets are being investigated due to its importance in future technological applications based on highly integrated devices [7]. Another hot topic in magnetism is the study of domain-wall dynamics in single nanowires [8], which is important for spintronic applications in general and, in particular, for magnetic domain-wall logic [9]. Consequently, the development of experimental techniques allowing a direct study of the magnetization reversal process in a single nanomagnet is of great importance from a fundamental and applied point of view. The magnetization reversal process of Co nanostructures has been characterized by magnetoresistance techniques [10] and spatially resolved magneto-optical Kerr effect (MOKE) [11]. However, the lateral resolution of the MOKE system (understanding the possibility to distinguish two adjacent elements) is limited by the laser spot diameter usually in the range of a few microns except in the case of magneto-optical scanning near-field optical microscopy [12]. Other techniques of high sensitivity as the Hall micromagnetometry [13] can be used to obtain the hysteresis loop of individual nanoparticles, although its use is limited in structures submitted to nanolithography. The variable field magnetic force microscopy (VF-MFM) [14] presented here is, therefore, more suitable for the study of highly dense nanostructures due to its high lateral resolution (20 nm although a resolution of 10 nm can be reached under special conditions as shown in Ref. [15]). This resolution is comparable to the one of the transmission techniques like Lorentz transmission electron microscopy [16] and STXM (scanning transmission x-ray microscopy) [17]; however, the MFM does not present the handicap of the sample thickness of the transmission samples.

## Experimental details

In the present work, we have studied by advanced MFM techniques the magnetization reversal of individual polycrystalline cobalt wires grown by focused electron-beam-induced deposition. The sample growth was performed in a commercial FEI dual-beam^® ^equipment using a field-emission scanning electron microscope with Co_2_(CO)_8 _as gas precursor, Si substrates and electron-beam conditions of 2.1 nA beam current, 10 keV beam energy, and 1 μs dwell time. As previously reported, a suitable set of growth parameters produce Co nanodeposits with high Co content (95%), magnetotransport properties similar to those of pure Co [18] and good domain-wall conduit behavior [19] The crystalline structure and the shape of the Co nanowires determine the effective magnetic anisotropy that is the balance between the magnetocrystalline and the shape anisotropies [20,21]. Notice that due to the polycrystalline character of the Co deposits, its magnetic behavior is controlled by the shape anisotropy instead of by magnetocrystalline anisotropy [11].

The measurements have been performed with a commercial magnetic force microscope from Nanotec Electronica S.L. (Madrid, Spain). This system has been adapted to apply *in situ *in-plane and out-of-plane magnetic fields [14]. Both mechanical and thermal stability of the system have been improved to prevent the relative tip-sample displacement during the field sweeping. In particular, the relative lateral displacement is about 0.014 nm/Oe when in-plane magnetic field is applied (more technical data in Ref. [14]). As a result, this system can be used to obtain high-resolution MFM images of individual nanostructures under continuously applied magnetic fields [22,23] or to study its magnetization reversal [24-26].

We have applied an MFM-based method [15,14] to perform local magnetic hysteresis loops of the wires in a novel way. This mode allows one to measure different magnitudes related to the tip-sample interaction (normal force, amplitude, phase, frequency shift, *etc*.) as a function of two parameters: the lateral displacement (X-scan) and the *in situ *applied magnetic field. In this particular case, a magnetic image (corresponding to the frequency shift mapping) is acquired along a surface profile (X-scan) as a function of the magnetic field (parallel to the X-scan direction). The tip is scanned along the main axis of the Co nanowire (which is the easy axis of the magnetization) about 30 nm above it. This method allows us to observe the evolution of the magnetic state *versus *the magnetic field of individual nanoelements. In previous works [27,28], the evolution of the frequency shift (or phase) *versus *the externally applied magnetic field is measured in different positions of the nanostructures. In the case of samples with low magnetic moment, other medium or long-range interactions between the tip and the sample can be significant [29]. The MFM imaging process of these materials can become more complex since the frequency shift (or the phase) depens drastically on the tip-sample configuration. Since in the method proposed here the tip is moving along the sample, its advantage is the high sensitivity to spurious changes in the tip-sample position. By using this new method, the probe performs every scan under a continuous magnetic field. Hence, we can evaluate the change in the MFM contrast while sweeping the magnetic field from scan to scan. In addition, the normal force and the amplitude of oscillation are simultaneously measured with the frequency shift in order to assure optimum feedback conditions. Thus, this MFM-based mode is a reproducible and fast method - the measuring time being less than 1 min.

For low-oscillation amplitude, the frequency shift of the cantilever - at a retrace distance enough to avoid Van der Waals interaction - is proportional to the value of the magnetic force gradient. In first approximation, the force gradient is proportional to the sample and tip magnetic moments [27]. Therefore, the changes of the frequency shift as a function of the external magnetic field can be used either to evaluate the coercivity of the MFM probes [15]^a ^or to analyze the magnetic behavior of micro and nanostructures [27,28] depending on the set of values of both the tip and sample coercitive fields (*H*_tip _and *H*_sample_) and the maximum field applied (*H*_max_). Since we are interested in the sample characterization, we will select as experimental conditions *H*_tip _>*H*_max _>*H*_sample _in order to avoid the depolarization of the tip.

The probes used in this experiment are commercial Si cantilevers (Nanosensors PPP-FMR, *k *= 1.5 N/m and *f *= 75 KHz) coated with a Co/Cr-sputtered thin film. The thickness of the coating (25 nm) has been selected in order to prevent the influence of the tip stray field on the magnetic state of the sample [30]. To guarantee the low in-plane stray field of the probes, the tips have been checked by imaging soft elements as Py dots. The behavior of these homemade tips under an externally applied magnetic field has been previously analysed [30] and the probes with higher coercive fields (about 450 Oe) than those of the Co nanostripes are chosen. Before each experiment, the probes are magnetized along their pyramid axis.

## Results and discussion

**Figure 1:**
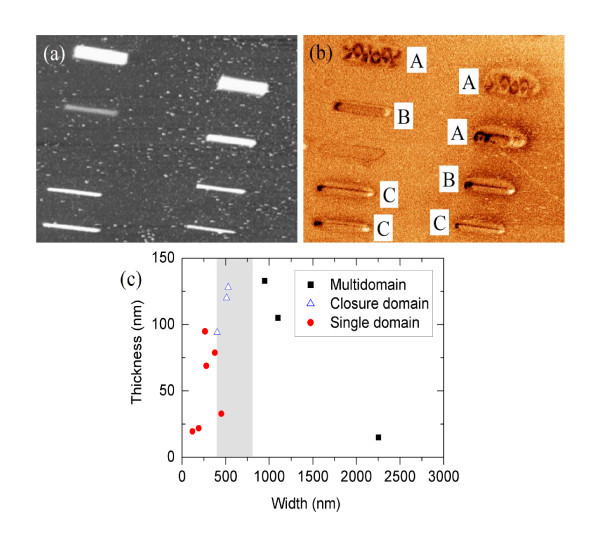
**Topography and magnetic image of a typical region of the sample**. (**a**) Topography and (**b**) MFM image of the array of nanowires (frequency shift contrast 11 Hz). Images size: 25.5 × 18.5 μm. Notice how the domain configuration is a function of the aspect ratio of the nanostructures (**c**) Nanowires domain configuration distribution as a function of their dimensions.

The MFM images of the whole array of Co nanowires measured in remanent state show the existence of different magnetic configuration as a function of the nanowire dimensions. The length of the nanowires is roughly constant (5.3 ± 0.1 μm) but the thickness varies between 20 to 140 nm and the width from 100 to 2,250 nm. The nanowires are well separated one from each other in order to avoid significant influence of dipolar interactions between nearby nanowires. Figure 1 displays the topography and the magnetic image of a typical region of the sample. Notice the evolution of the magnetic configuration from a multidomain structure (labeled A) to a single-domain state (labeled C). The largest nanostructures exhibit multidomain configuration in good agreement with magnetoresistance measurements on cobalt wires patterned by electron-beam lithography [31]. Nanowires narrower than 400 nm present single-domain state. In between, we distinguish nanostructures with rather complicated closure domain structure in their extremes (labeled B). Figure 1c shows the distribution of these three different configurations regarding the nanostructure width. For the wire dimensions reported here, the magnetization reversal is not expected to be influenced by structural defects. As previously discussed in Ref. [11], micromagnetic simulations support that the shape anisotropy is able to explain the main features of the magnetization reversal in this type of wires with dimensions around 200 nm in width. Thus, domain-wall pinning effects caused by structural defects are not expected for the wire dimensions studied here (width of 400 nm or larger) but only for much narrower wires.

For a better understanding of the results, we present here the detailed results corresponding to the behavior of two kinds of nanowires with thickness around 100 nm but different width.

**Figure 2:**
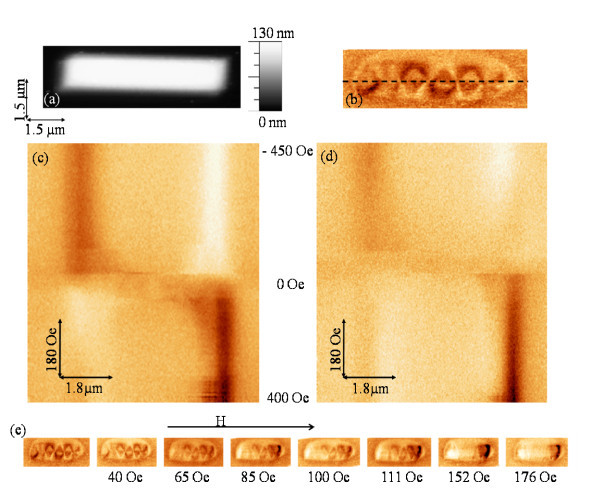
**Topography and MFM image of a 1-μm-width nanowire (type A)**. (**a**) Topography and (**b**) in-remanence MFM image of nanowire A; (**c**)**-**(**d**) MFM-based mode images obtained along the dashed line in (b); (**e**) MFM images under different *in situ *magnetic field. The frequency shift contrast for all the MFM images is 5 Hz.

Figure 2 shows the topography (a) and the MFM image (b) of a 1-μm-width nanowire (type A). The magnetic image has been measured in remanence after saturating the sample along its easy axis under an *ex situ *field of 5 kOe. The magnetic configuration is a complex multidomain structure with multiples vortices. We have performed the MFM-based mode measurements where the tip scans along the dashed line drawn in Figure 2b, located at the center of the wire, while increasing (Figure 2c) or decreasing (Figure 2d) the in-plane magnetic field applied along the *x *direction. The signal shown in Figure 2c, d corresponds to the frequency shift that is proportional to the magnetic force gradient. The vertical axis in these images is the external magnetic field. In Figure 2c, the magnetic field increases - as shown by the arrow - from -450 Oe (top of the image) to +400 Oe (at the bottom of the image). The subsequent image Figure 2d begins when the magnetic field starts to decrease from +400 Oe (corresponding to the scan at the bottom of the image) to −450 Oe (reached in the top scan).

Those magnetic images show clearly the two saturation states at the maximum magnetic fields. For low magnetic field values (near zero) the nanostructure develops a complex domain configuration. The single-domain state is reached again for 250 Oe. In the MFM images series presented in Figure 2e, the magnetization reversal process of the structure can be followed.

**Figure 3:**
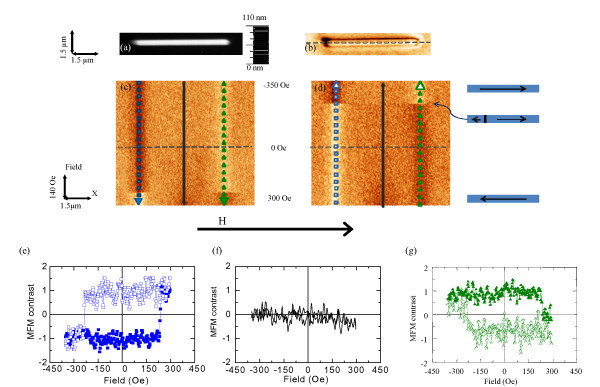
**Topography and in-remanence magnetic image of a single-domain nanowire (type C)**. (**a**) Topography and (**b**) MFM image in remanence of nanowire C; (**c**)**-**(**d**) MFM-based mode images (**e**)**-**(**g**) profiles corresponding to hysteresis loops. The frequency shift contrast for all the MFM images is 8.5 Hz.

Different behavior is observed in narrower wires (labeled C). Figure 3a, b correspond respectively to the topography and in-remanence magnetic image of a single-domain nanowire 260 nm width. Using this MFM-based mode, we have measured the magnetic signal along the dashed line drawn in Figure 3b while increasing (Figure 3c) or decreasing (Figure 3d) the in-plane magnetic fields applied along the *x *direction. The *Y *scale in the images is the external magnetic field that is increased (decreased) at each scan line from -350 Oe at the upper line to 300 Oe at the lowest scan line.

In both images, a clear change in the frequency shift contrast is observed when the magnetic field is around 240 Oe. This critical field corresponds to changes in the nanostructure domain configuration. Measuring the contrast along the profiles marked with square and triangular dots in Figure 3c, d, the hysteresis loop of the nanostripe is obtained (see Figure 3e, f, g). The coercive field of this particular nanostructure can be deduced from the hysteresis loops measured on the edges of the nanostripe (Figure 3e, g). Notice that no changes are observed in the profiles (solid line) performed along the middle of the Co nanostructure (shown in Figure 3f). This is expected because the domain-wall velocity during magnetization reversal is very fast compared to the image acquisition time.

## Conclusions

In summary, the use of advanced methods in MFM allows us to gain information about the magnetization reversal process in nanostructures. The MFM-based technique presented in this work has two important values: it is much faster than to measure a collection of images at different magnetic fields (at least 100 times) and allows us to display continuously the magnetization reversal process. This method provides the individual coercive field of single-domain nanostructures. Since the MFM contrast can be measured as the magnetic field varies continuously, this methodology can be used to characterize different critical fields as the nucleation and annihilation fields in magnetic dots which present vortex configuration. Notice that the study of individual elements is not possible with macroscopic - although high sensitive - techniques like SQUID or VSM and thus the VF-MFM working in this new mode arises as a promising local magnetometer. Moreover, since the critical fields of individual elements can be evaluated simultaneously with its topography, it is possible to perform statistical studies about the relationship between the magnetic behavior and the topographic features.

## Competing interests

The authors declare that they have no competing interests.

## Authors' contributions

AA and JMDT conceived of the collaborative study and coordinated it. MJ and AA conceived of the new MFM technique to characterize the coercive field of individual nanostructures. JMDT, LSR, AFP and MRI defined the geometry and the composition of the nanowire arrays. LSR grew the samples and participated in the magnetic characterization. MJ and OIF carried out the magnetic characterization by MFM. All the authors discussed the results, contributed to the manuscript text and approved its final version.

## Endnotes

^a^However, the use of this method is limited for soft materials since a systematic error could appear in the evaluation of the coercive field due to the stray field of the tip
